# Concatenated Composite Pulses Applied to Liquid-State Nuclear Magnetic Resonance Spectroscopy

**DOI:** 10.1038/s41598-020-58823-9

**Published:** 2020-02-07

**Authors:** Masamitsu Bando, Tsubasa Ichikawa, Yasushi Kondo, Nobuaki Nemoto, Mikio Nakahara, Yutaka Shikano

**Affiliations:** 1 Kindai University Technical College, 7-1 Kasugaoaka, Nabari, Mie 518-0459 Japan; 2 Department of Physics, Gakushuin University, 1-5-1 Mejiro, Toshima-ku, Tokyo, 171-8588 Japan; 3 Research Center for Quantum Computing, Interdisciplinary Graduate School of Science and Engineering, Kindai University, 3-4-1 Kowakae, Higashi-Osaka, Osaka, 577-8502 Japan; 4 Department of Physics, Kindai University, 3-4-1 Kowakae, Higashi-Osaka, Osaka, 577-8502 Japan; 5 Research Institute for Science and Technology, Kindai University, 3-4-1 Kowakae, Higashi-Osaka, Osaka, 577-8502 Japan; 6 JEOL RESONANCE Inc., 3-1-2 Musashino, Akishima, Tokyo, 196-8558 Japan; 7 Department of Mathematics, Shanghai University, 99 Shangda Road, Shanghai, 200444 China; 8 Quantum Computing Center, Keio University, 3-14-1 Hiyoshi, Yokohama, Kanagawa, 223-8522 Japan; 9 Institute for Quantum Studies, Chapman University, 1 University Dr., Orange, CA 92866 USA; 10 Research Center of Integrative Molecular Systems (CIMoS), Institute for Molecular Science, National Institutes of Natural Sciences, 38 Nishigo-Naka, Myodaiji, Okazaki, 444-8585 Japan; 11 Materials and Structures Laboratory, Tokyo Institute of Technology, 4259 Nagatsuta, Midori, Yokohama, Kanagawa 226-8503 Japan; 12 Research Center for Advanced Science and Technology (RCAST), The University of Tokyo, 4-6-1 Komaba, Meguro, Tokyo, 153-8904 Japan

**Keywords:** Quantum information, Quantum metrology

## Abstract

The error-robust and short composite operations named ConCatenated Composite Pulses (CCCPs), developed as high-precision unitary operations in quantum information processing (QIP), are derived from composite pulses widely employed in nuclear magnetic resonance (NMR). CCCPs simultaneously compensate for two types of systematic errors, which was not possible with the known composite pulses in NMR. Our experiments demonstrate that CCCPs are powerful and versatile tools not only in QIP but also in NMR.

## Introduction

Nuclear magnetic resonance (NMR) is widely used for chemical analysis of various molecules by pharmaceutical companies^1^ owing to highly developed NMR techniques^2^. Some of these advanced techniques in NMR have been transferred to quantum information processing (QIP)^3^ because NMR manipulations are regarded as controlling and measuring quantum objects, called spins. We have been working on transferring one of the existing NMR techniques, a composite pulse^4–7^ that realises a reliable single spin rotation with erroneous pulses, to QIP. There are two types of composite pulses in NMR: One compensates for pulse-length errors (PLEs), whereas the other compensates for off-resonance errors (OREs). PLEs correspond to rotation angle errors in the dynamics of the qubit on a Bloch sphere, and OREs to rotation axis errors. We have successfully developed an error-robust and short-pulse-length composite operation (pulses), named ConCatenated Composite Pulses (CCCPs), by combining the above-mentioned two types of composite pulses in an effort to develop high-precision unitary operations^8,9^ for QIP. This third type of composite pulses simultaneously compensates for the two types of errors (PLEs and OREs in NMR) at the cost of operation time, which was not possible with the known composite pulses in NMR.

The purpose of this paper is to feedback our achievement for QIP to NMR. CCCPs are able to lead significant signal strength improvement without any changes in the hardware settings.

Let us briefly review the principle of composite pulses compensating PLEs or OREs in NMR^6^. Throughout this paper, the system is a nucleus with spin 1/2 (in short, a *spin*) in a static magnetic field along the *z*-axis. An ideal rotation operation of the spin without errors is given as

1 $$R(\theta ,\phi )=\exp [-{\rm{i}}\theta {\boldsymbol{n}}(\phi )\cdot {\boldsymbol{\sigma }}/2],$$

where *θ* is the rotation angle, ${\boldsymbol{n}}(\phi )=(\cos \phi ,\sin \phi ,0)$ is the rotation axis in the *xy*-plane, and ***σ*** = (*σ*_*x*_, *σ*_*y*_, *σ*_*z*_) is the Pauli matrices. This rotation may be realized by a radio-frequency pulse in NMR, the frequency of which is the same as the Larmor frequency of the spin.

We consider a realistic pulse in which a PLE and/or an ORE are present. The first-order terms of the errors are discussed since we are interested in the cases where the errors are small. The rotation operator ${R^{\prime} }_{\varepsilon }(\theta ,\phi )$ associated with a pulse under a PLE is given as

2 $$\begin{array}{ccc}{R^{\prime} }_{\varepsilon }(\theta ,\phi )=\exp [-{\rm{i}}(1+\varepsilon )\theta {\boldsymbol{n}}\cdot {\boldsymbol{\sigma }}/2]=R(\theta ,\phi )-{\rm{i}}\varepsilon \theta ({\boldsymbol{n}}\cdot {\boldsymbol{\sigma }})R(\theta ,\phi )/2, &  & \end{array}$$

where *ε* is the strength of the PLE, which is unknown — but constant and small. Higher-order terms beyond the first order in *ε* are suppressed in the second equality. This type of error cannot be avoided because of inhomogeneity in the *B*_1_ field^10^. By contrast, the rotation operator ${R}_{f}^{^{\prime} }(\theta ,\phi )$ associated with a pulse under an ORE is given as

3 $$\begin{array}{ccc}{R^{\prime} }_{f}(\theta ,\phi )=\exp \left[-{\rm{i}}\theta ({\boldsymbol{n}}\cdot {\boldsymbol{\sigma }}+f{\sigma }_{z})/2\right]=R(\theta ,\phi )-{\rm{i}}f\sin (\theta /2){\sigma }_{z}, &  & \end{array}$$

where *f* is the strength of the ORE. OREs are caused whenever the Larmor frequency of the spin is not the same as the transmitter frequency. Therefore, OREs cannot be avoided in NMR measurements because of the chemical shifts of spins. As with a PLE, *f* is unknown — but constant and small. Therefore, when both a PLE and an ORE are present, the rotation associated with a pulse is given as

4 $$\begin{array}{lll}R^{\prime} (\theta ,\phi ) & = & \exp \left[-{\rm{i}}(1+\varepsilon )\theta ({\boldsymbol{n}}\cdot {\boldsymbol{\sigma }}+f{\sigma }_{z})/2\right]\\  & = & R(\theta ,\phi )-{\rm{i}}\varepsilon \theta ({\boldsymbol{n}}\cdot {\boldsymbol{\sigma }})R(\theta ,\phi )/2-{\rm{i}}f\sin (\theta /2){\sigma }_{z}.\end{array}$$

The second line is an approximation when both *ε* and *f* are small.

The NMR community has developed a technique to overcome PLEs or OREs by combining several pulses^3,6,7^. Given a target rotation *R*(*θ*, *ϕ*), we can find an equivalent rotation sequence that is equal to the target *R*(*θ*, *ϕ*) in a case without errors, as follows:

5 $$R({\theta }_{N},{\phi }_{N})R({\theta }_{N-1},{\phi }_{N-1})\cdots R({\theta }_{1},{\phi }_{1})=R(\theta ,\phi ).$$

Here, *R*(*θ*_*i*_, *ϕ*_*i*_) is the *i*-th rotation associated with the *i*-th pulse, and *N* denotes the number of pulses. The point of the decomposition (5) is

6 $${R}^{^{\prime} }({\theta }_{N},{\phi }_{N}){R}^{^{\prime} }({\theta }_{N-1},{\phi }_{N-1})\cdots {R}^{^{\prime} }({\theta }_{1},{\phi }_{1})\ne {R}^{^{\prime} }(\theta ,\phi )$$

if a PLE and/or an ORE exist. This non-equality is caused by the non-commutativity among *R*(*θ*_*i*_, *ϕ*_*i*_). Therefore, by appropriately tuning the parameters ${\{{\theta }_{i},{\phi }_{i}\}}_{i=1}^{N}$ in Eq. (5), we may be able to obtain a sequence that (i) virtually works as the target *R*(*θ*, *ϕ*) when there are no errors, and (ii) is less sensitive to the systematic errors. Indeed, various pulse sequences have been designed^4–6,11–14^ in such a way that Eq. (5) has no first-order terms of errors if only one of *ε* and *f* exists^6^. We state that such a pulse sequence without the first-order term of *ε* (*f*) is *r*obust against PLEs (OREs).

We now present two typical composite pulses that are robust against either PLEs or OREs: Broad Band 1 (BB1)^11^, and Compensation for Off-Resonance with a Pulse SEquence (CORPSE)^12^. See more details in Methods. BB1 is designed in order to compensate for a PLE and behaves as

7 $${R}_{{\rm{BB1}}}^{^{\prime} }(\theta ,\phi )=R(\theta ,\phi )-{\rm{i}}f\sin (\theta /2){\sigma }_{z},$$

under both a PLE and an ORE. BB1 filters out the PLE but leaves the ORE unchanged, which we call the residual error preserving property (REPP) with respect to ORE. In contrast to BB1, CORPSE is a composite pulse robust against OREs and behaves as

8 $${R}_{{\rm{CORPSE}}}^{^{\prime} }(\theta ,\phi )=R(\theta ,\phi )-{\rm{i}}\varepsilon ({\boldsymbol{n}}\cdot {\boldsymbol{\sigma }})R(\theta ,\phi )/2.$$

Thus, CORPSE possesses REPP with respect to PLE. Not all composite pulses have REPP, which was not known before ref. ^15^.

We show how to design a CCCP that compensates for both a PLE and an ORE simultaneously by taking advantage of REPP, with BB1 and CORPSE as an example^15,16^. BB1 is robust against PLEs, and CORPSE is robust against OREs and has the REPP with respect to the PLE. Therefore, we replace all pulses in BB1 with CORPSE. This CCCP is called CORPSE-in-BB1, or CinBB in short. The number of pulses in CinBB is 4 × 3 = 12. The number of pulses in CinBB can be further reduced to *N* = 6, and the resulting CCCP is called the reduced CinBB (R-CinBB). See Methods and ref. ^15^ for further details. Another interesting approach to tackle both PLEs and OREs was discussed by Jones^17^, in which composite pulses were designed to compensate for higher-order error terms of both PLEs and OREs simultaneously. The rotation angle *θ* is, however, fixed to *π* in these composite pulses. See the review by Merrill and Brown^18^ on composite pulses including CCCPs.

**Figure 1:**
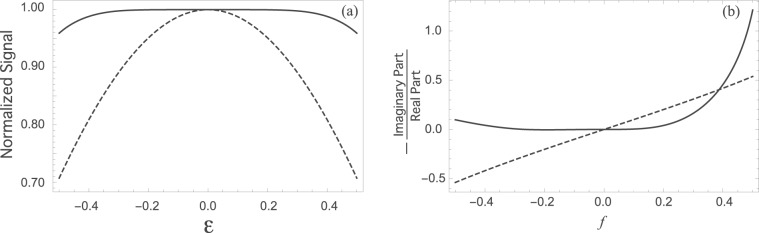
The effect of error size on signal. (**a**) Normalized signal amplitude after a *π*/2-pulse. The dashed (solid) curve is the outcome of a single square (R-CinBB) pulse as a function of *ε* (the strength of the pulse length error). (**b**) $-\frac{{\rm{Imaginary}}\ {\rm{Part}}}{{\rm{Real}}\ {\rm{Part}}}$, a measure of the direction error from the *x*-axis (the direction of the magnetization without errors), is plotted as a function of *f* (the strength of the off-resonance error). The calculations with Eq. (4) are performed without approximation because $\left|\varepsilon \right| \sim 0.5$ and $\left|f\right| \sim 0.5$ cannot be regarded as small.

The signal after a single square *π*/2-pulse is shown as the dashed lines in Fig. 1. This single square pulse has a constant *B*_1_ during the period of *τ*_*p*_ and *B*_1_*τ*_*p*_ is *π*/2. Its rotation axis in the Bloch sphere is, here, the *y*-axis, and thus the magnetization after the *π*/2-pulse is in parallel to the *x*-axis if there are no errors. Figure 1(a) shows the normalized signal as a function of *ε* (the dotted curve); *ε* as small as *ε* = 0.1 leads to a significant signal reduction. Figure 1(b) shows that the magnetization after the single square *π*/2-pulse deviates from the *x*-axis and its deviation appears to be proportional to *f*. Then, let us consider the signal after the R-CinBB *π*/2-pulse which consists of six square pulses (see Methods for details). The solid line in Fig. 1(a) shows that one obtains a larger signal for a wide range of *ε* with the R-CinBB *π*/2-pulse than with the single square *π*/2-pulse. On the other hand, the solid line in Fig. 1(b) shows that the magnetization after the R-CinBB *π*/2-pulse is close to the *x*-axis for a wider range of *f* than after the single square *π*/2-pulse.

## Results

### Simulations of NMR experiments

Let us take into account a non-unitary time development caused by a spin–spin relaxation with a characteristic time *T*_2_ in simulating NMR experiments. We introduce this effect as a phase flip channel^19^. In the case of single-spin experiments,

9 $$\rho (t+\Delta )={p}_{ss}(\Delta )\rho (t)+(1-{p}_{ss}(\Delta )){\rm{Ad}}({\sigma }_{z},\rho (t)),$$

where ${p}_{ss}(\Delta )=(1+\exp (-\Delta /{T}_{2}))/2\approx 1-\Delta /2{T}_{2}$ and Ad(*ξ*, *ρ*) = *ξ*^†^*ρξ* with an arbitrary unitary operator *ξ*. Δ is a small time interval. The subscript *ss* denotes "spin-spin”.

The time evolution during a pulse is simulated as follows:

10 $$\begin{array}{ll}\widetilde{\rho }(t+{\tau }_{p}) & =\,{p}_{ss}({\tau }_{p})\rho (t)+\left(1-{p}_{ss}({\tau }_{p})\right){\rm{Ad}}({\sigma }_{z},\rho (t)),\\ \rho (t+{\tau }_{p}) & =\,{\rm{Ad}}({U}_{{\rm{pulse}}},\widetilde{\rho }(t+{\tau }_{p})),\end{array}$$

where *U*_pulse_ is a unitary operation generated by the pulse. Note that *τ*_*p*_ is the total pulse duration and is assumed to be small. Therefore, we employ the Suzuki-Trotter formula, which ensures the decomposition of the dynamical evolution into the form of pure relaxation process followed by the application of the composite pulse^20^.

**Figure 2:**
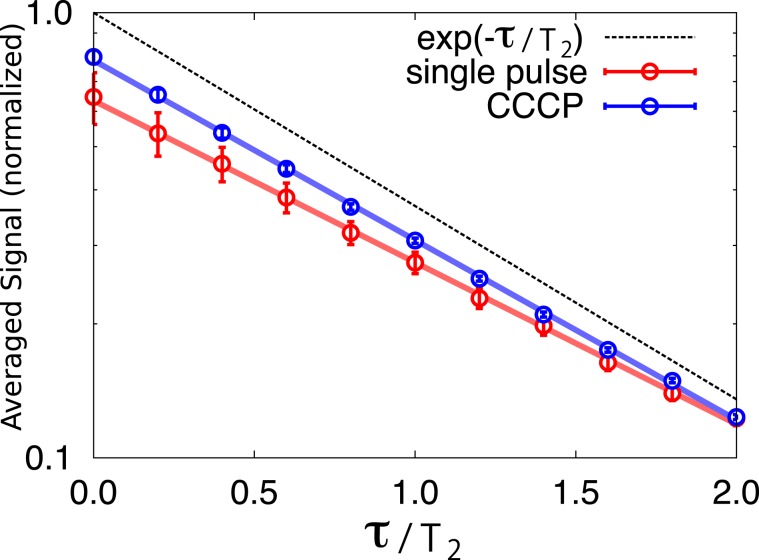
Semi-log plot of echo signals with two square (red line) and two R-CinBB (blue line) pulses as functions of the waiting time *τ*. The black dashed line is an error-free case. Error bars represent the fluctuation of the signal strength. The figure shows that the R-CinBB pulses suppress the fluctuation of the echo signals.

**Figure 3:**
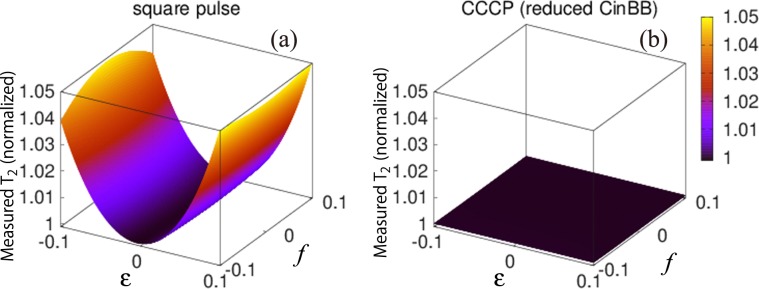
Measured *T*_2_ as a function of a PLE and an ORE for (**a**) square pulses and (**b**) R-CinBB pulses. The Hahn echo experiments with R-CinBB pulses lead to the correct *T*_2_, even in erroneous cases. Measured *T*_2_’s are normalized by the true *T*_2_.

We examine Hahn echo experiments^2^ with two pulses which are affected by fluctuating PLEs and OREs. Their means are $\bar{\varepsilon }=\bar{f}=0.1$ and their standard deviations are both 0.08. Although these values may be unreasonably large for modern NMR spectrometers, simulated results show that the echo signals with R-CinBB pulses do not fluctuate, as shown in Fig. 2. Simulations of the Hahn echo experiments as a function of the error strengths are summarized in Fig. 3. The Hahn echo experiments with two single square pulses (Fig. 3a) are strongly affected by PLEs, whereas those with R-CinBB pulses are robust against these errors (Fig. 3b). It turns out that a composite pulse robust against PLEs is sufficient for obtaining a correct *T*_2_ even when both PLEs and OREs are present.

Let us examine two-dimensional (2D) shift-COrrelation SpectroscopY (COSY) experiments, one of the most important NMR measurement methods^1^, with two interacting spins. The interaction is a scalar coupling in a weak coupling limit^2^. The simulations during the evolution and detection periods^1^ are done as follows:

11 $$\begin{array}{ll}\widetilde{\rho }(t+\delta ) & =\,\left(1-\frac{\delta }{2{T}_{2,1}}-\frac{\delta }{2{T}_{2,2}}\right)\rho (t)+\frac{\delta }{2{T}_{2,1}}{\rm{Ad}}({\sigma }_{z}\otimes {\sigma }_{0},\rho (t))+\frac{\delta }{2{T}_{2,2}}{\rm{Ad}}({\sigma }_{0}\otimes {\sigma }_{z},\rho (t)),\\ \rho (t+\delta ) & =\,{\rm{Ad}}\left(\exp \left(-J\delta \frac{{\sigma }_{z}\otimes {\sigma }_{z}}{4}\right),\widetilde{\rho }(t+\delta )\right),\end{array}$$

where *T*_2,*i*_ is the spin-spin relaxation time of the *i*-th spin. Equation (11) is again, similarly to Eq. (10), based on the Suzuki-Trotter formula^20^: The first equation in Eq. (11) describes the spin-spin relaxation channel and the second one is the time development generated by the spin-spin interaction.

**Figure 4:**
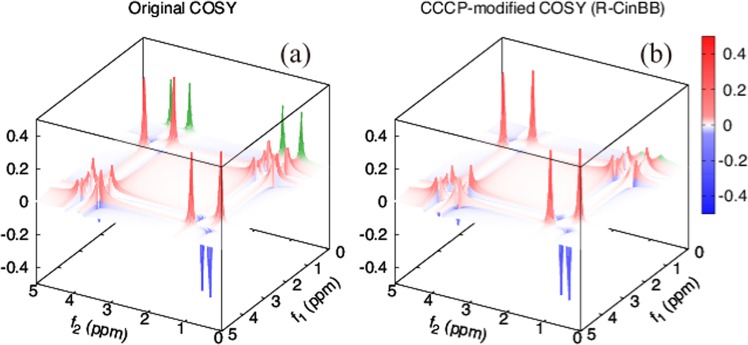
Simulation of COSY experiments of a two-spin molecule with (**a**) two single square and (**b**) R-CinBB pulses, where *ε* = *f* = 0.1, *J* = 0.5 ppm, and chemical shifts are 1 and 4 ppm. Spurious axial (green) peaks are observed in the simulation with square pulses, whereas no such peaks are observed in that with R-CinBB pulses.

*ρ*(*t* + *nδ*) can be obtained by iterating the above operations *n* times. Note that *p*_*ss*_(*δ*) ≈ 1 − *δ*/(2*T*_2,*i*_) for the *i*-th spin because *δ* is sufficiently small compared to *T*_2,*i*_. During a pulse, the time development is simulated similarly to the case of single-spin experiments. Simulations are summarized in Fig. 4 in the case that the chemical shifts of these spins are 1 and 4 ppm and *J* = 0.5 ppm. In COSY experiments, spurious peaks called axial peaks sometimes appear owing to the inaccuracy of the first pulse^1^. We are able to reproduce these axial peaks in the simulation of the COSY experiments with two single square pulses (*ε* = *f* = 0.1), as shown in Fig. 4a. By contrast, no axial peaks appear in the simulation with R-CinBB pulses in Fig. 4b.

### Experimental demonstration of NMR measurements with CCCPs

**Figure 5:**
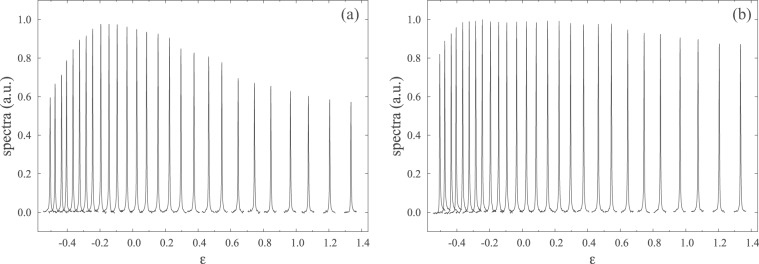
Series of 1D spectra with (**a**) square *π*/2-pulse and (**b**) R-CinBB *π*/2-pulse applied as functions of the PLE (*ε* in (Eq. (4))). The strength of the ORE is fixed at *f* ~ 0.

**Figure 6:**
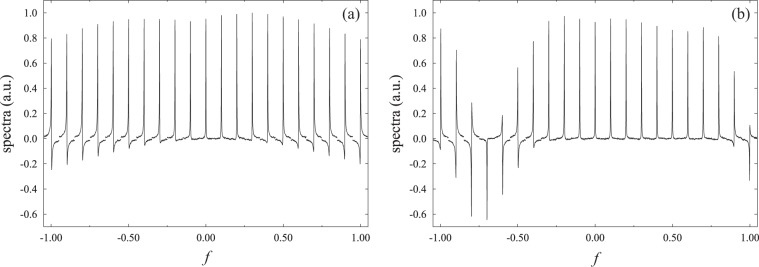
Series of 1D spectra with (**a**) square *π*/2-pulse and (**b**) R-CinBB *π*/2-pulse applied as functions of the ORE (*f* in Eq. (4)). The strength of the PLE is fixed at *ε* ~ 0.

The advantages of CCCPs in NMR are demonstrated in the following experiments. The single-pulse experiments were carried out using 300 mM ^13^C-labelled chloroform in acetone-*d*_6_ at 25 °C. We examined the performance of a composite *π*/2-pulse applied to ^13^C and compared the result with that of a single square pulse^21^. It is clear that the R-CinBB pulses are more advantageous than single square pulses in terms of PLE, as shown in Fig. 5. This is also demonstrated in the corresponding numerical calculations, shown in Fig. 1(a). We also examined the R-CinBB pulse in terms of the ORE, as shown in Fig. 6 (see also Fig. 1(b)). The R-CinBB composite pulse is clearly more advantageous than the square pulse when −0.3 < *f* < 0.8. Although the spectra with the R-CinBB composite pulses corresponding to *f* < −0.5 and 1.0 < *f* are more distorted than those of the single square pulses, such large *f*   ’s are not relevant in usual experiments. See ref. ^15^ for details.

**Figure 7:**
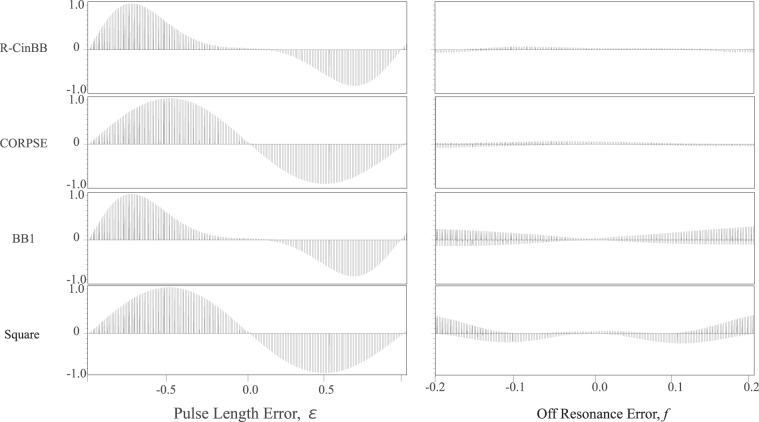
Series of 1D ^1^H-NMR spectra of 2% HDO solution measured after two successive (R-CinBB, CORPSE, BB1, and square) *π*/2-pulses. All the spectra shown in these eight panels are normalized with the signal intensity obtained by a single square *π*/2-pulse spectrum (data not shown). The single square *π*/2-pulse duration is 9.95 *μ*s. Each panel contains 201 1D spectra.

The advantage of the R-CinBB *π*/2-pulse in NMR is also evaluated, as shown in Fig. 7. We applied two successive (R-CinBB, CORPSE, BB1, or square) *π*/2-pulses to the thermal equilibrium state. A pair of successive *π*/2-pulses is equivalent to a single *π*-pulse without errors and should lead to no signal. Therefore, the observed residual signals are measures of errors in these pulses. The advantage of the R-CinBB *π*/2-pulse is clear from the fact that the two successive R-CinBB *π*/2-pulses lead to small signals in wider ranges of both PLEs (*ε*) and OREs (*f*   ).

**Figure 8:**
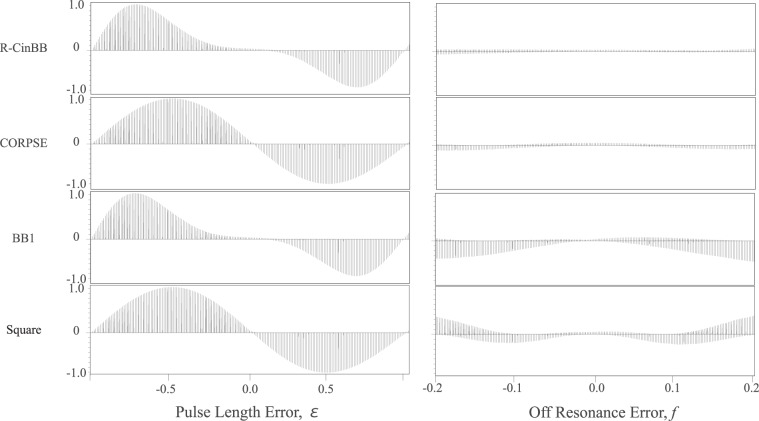
Series of 1D ^1^H-NMR spectra of 2% HDO solution measured after single (R-CinBB, CORPSE, BB1, and square) *π*-pulses. All the spectra shown in these eight panels are normalized with the signal intensity obtained by the single square *π*/2-pulse spectrum used in Fig. 7. Each panel contains 201 1D spectra.

The advantage of the R-CinBB pulse in NMR is also evaluated in the case of *π*-pulses, as shown in Fig. 8. We applied a (R-CinBB, CORPSE, BB1, or square) *π*-pulse to the thermal equilibrium state. An ideal *π* pulse should lead to no signal. The advantage of the *π* R-CinBB pulse is clear from the fact that the R-CinBB *π*-pulses lead to small signals in wider ranges of both PLEs (*ε*) and OREs (*f*). These experiments were carried out as in the case of Fig. 7. It is interesting to note that the behaviours as a function of *ε* of CORPSE and square pulses are identical, which indicates that CORPSE has REPP with respect to PLE in the whole range of *ε* in Figs. 7 and 8. On the other hand, the REPP of BB1 with respect to ORE is only valid for small $\left|f\right|$.

**Figure 9:**
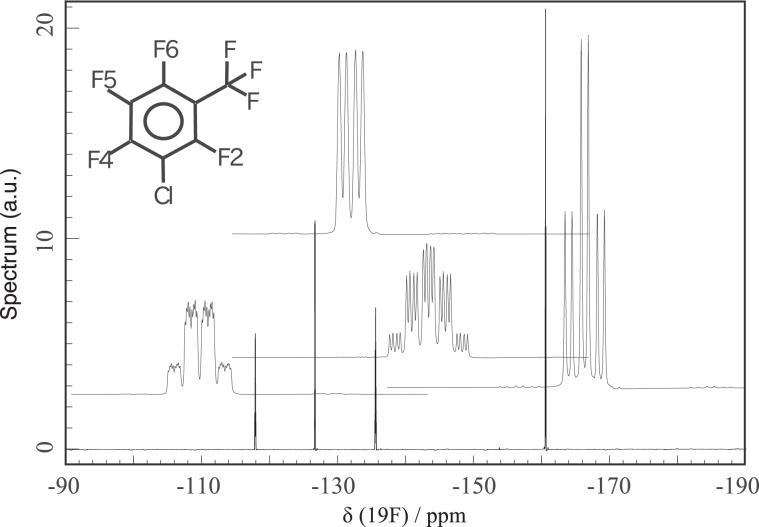
1D ^19^F-NMR spectrum of 300 mM 3-chloro-2,4,5,6-tetrafluoro-benzotrifluoride in benzene-*d*_6_. Each peak is enlarged to show detailed structures (1 ppm width). Peak assignments are as follows: 1 (−118.0 ppm), 2 (−126.5 ppm), 3 (−135.5 ppm), and 4 (−160.5 ppm) are identified as F2, F4, F6, and F5, respectively. The ^19^F signal of the trifluoromethyl group is not observed in this frequency region.

Next, we performed COSY experiments of 300 mM 3-chloro-2,4,5,6-tetrafluoro-benzotrifluoride in benzene-*d*_6_. We utilized ^19^F at 2, 4, 5, and 6 as the target nuclear spin. *T*_1_’s are between 0.6 and 1.0 s, whereas *T*_2_’s are ~0.3 s. We chose this molecule for the following reasons. First, ^19^F signals of the molecule are widely spread, as shown in Fig. 9. Second, the spectrum pattern is complex enough to examine the performance of the pulses, despite of its simple molecular structure.

Here, the pulse duration of a single square *π*/2-pulse is 12.4 μs, which corresponds to a *B*_1_ strength of 20 kHz in frequency. The total duration of the R-CinBB pulse is 16.1 × 12.4 μs = 2.00 × 10^2^ μ*s*, which is almost instantaneous compared with the inverse of the interaction strength in frequency. Therefore, the replacement of a square pulse with the R-CinBB pulse should not cause problems for most applications of liquid state NMR measurements.

**Figure 10:**
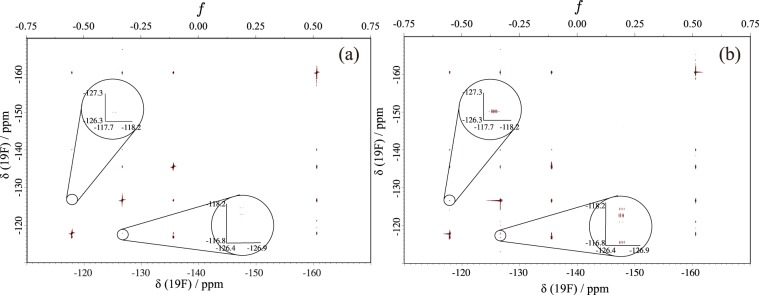
^19^F-^19^F COSY spectra of 300 mM 3-chloro-2,4,5,6-tetrafluoro-benzotrifluoride in benzene-*d*_6_ obtained with (**a**) two successive single square pulses and (**b**) R-CinBB pulses. The regions −118.0 ppm(*f*_1_) / −126.5 ppm(*f*_2_) and −126.5 ppm(*f*_1_) / −118.0 ppm(*f*_2_) are enlarged.

**Figure 11:**
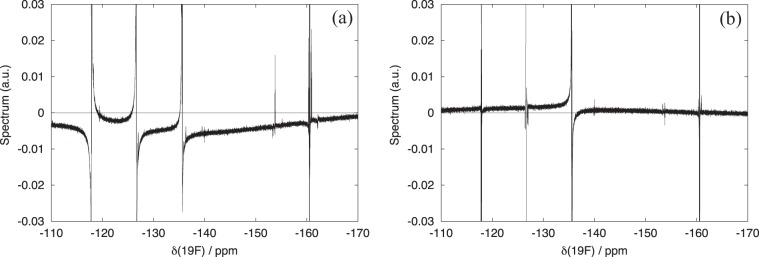
First increments of the ^19^F-^19^F COSY obtained with (**a**) square pulses and (**b**) R-CinBB pulses (2D spectra are shown in Fig. 10).

Since the *B*_1_ strength is 20 kHz, it is comparable to the frequency difference between the highest (−160 ppm) and the lowest (−118 ppm) peaks at 11.7 T (488 MHz for ^19^F and 500 MHz for ^1^H); see Fig. 9. In the case of square pulses, the correlation peak between −118.0 ppm (*f*_1_) and −126.5 ppm (*f*_2_), and that between −126.5 ppm (*f*_1_) and −118.0 ppm (*f*_2_), are hardly visible. As shown in Fig. 10, however, these have much higher intensities in the case of the R-CinBB pulses. In addition, the advantage of the R-CinBB pulses is much more clearly demonstrated in the one-dimensional (1D) spectra in Fig. 11. The phases of peaks obtained with square pulses are highly distorted. This may be one of the biggest reasons why the above correlation peaks are almost invisible.

## Discussion

Composite pulses have been developed in the NMR community and are widely employed. Our proposed composite operations, CCCPs, directly descend from these and have been developed as robust unitary operations for QIP. We feedback our achievements to NMR: We applied CCCPs to liquid-state NMR spectroscopy and demonstrated improved NMR sensitivity compared to standard 1D and 2D NMR measurements with square pulses. The proposed CCCPs are robust against two systematic errors, the PLE and ORE in NMR, at the cost of execution time.

We demonstrated the advantage of the R-CinBB pulses over the BB1, CORPSE, and square pulses in 1D and 2D (COSY) experiments. In terms of the compensation of PLEs and OREs, the replacement of single square pulses with CCCPs, such as the R-CinBB pulses, should be widely utilized in other experiments in liquid-state NMR. On the other hand, the application to solid-state NMR (SS-NMR) may be limited, because shorter pulses are favourable in SS-NMR in general and much longer CCCPs might be unacceptable in most cases. In the case of SS-NMR, COM-I, II, and III pulses^22^ are often employed as wideband (robust against *f*) pulses. These pulses employ only 0° and 180° phase pulses and thus the requirement of the electronics is less demanding compared with our proposed CCCPs. We believe, however, that advances in electronics can now allow use of CCCPs even in SS-NMR experiments.

CCCPs consist of simple spin rotation pulses and thus they are technically easy to implement although they are not optimal in terms of quantum control theory^23–26^. As mentioned before, composite pulses are widely used in NMR experiments, and we hope that CCCPs will be employed instead of these known composite or single square pulses because of their advantage. We believe that CCCPs should be useful for magnetic resonance imaging, too. Furthermore, CCCPs might be applied to positron *g*/2 measurements through the use of a ^3^He-NMR probe^27^ in which the inhomogeneity of an excitation field may be large. Also, because the pulse sequence in nonlinear optical spectroscopy has been inspired by the NMR pulse techniques^28^, CCCPs may be applicable in such optical systems in order to enhance the accuracy of optical spectroscopy.

## Material and Methods

### BB1

BB1^11^ is an *N* = 4 composite pulse robust against PLEs. The parameters are as follows:

12 $${\theta }_{1}={\theta }_{3}=\pi ,\,{\theta }_{2}=2\pi ,\,{\theta }_{4}=\theta ,{\phi }_{1}={\phi }_{3}=\phi +\arccos [-\theta /(4\pi )],\,{\phi }_{2}=3{\phi }_{1}-2\phi ,\,{\phi }_{4}=\phi .$$

BB1 under both a PLE and an ORE results in

13 $${R^{\prime} }_{{\rm{B}}B1}(\theta ,\phi )=R(\theta ,\phi )-{\rm{i}}f\sin (\theta /2){\sigma }_{z},$$

### CORPSE

CORPSE^12^ is an *N* = 3 composite pulse robust against OREs. Its parameters are

14 $$\begin{array}{rcl}{\theta }_{1} & = & 2{n}_{1}\pi +\theta /2-k,\\ {\theta }_{2} & = & 2{n}_{2}\pi -2k,\\ {\theta }_{3} & = & 2{n}_{3}\pi +\theta /2-k,\\ {\phi }_{1} & = & {\phi }_{2}-\pi ={\phi }_{3}=\phi ,\\ k & = & \arcsin [\sin (\theta /2)/2],\end{array}$$

where *n*_1_, *n*_2_, and *n*_3_ are non-negative integers. In particular, when we take *n*_1_ = *n*_3_ = 0 and *n*_2_ = 1, the execution time is minimized. In this case, CORPSE is referred to as short CORPSE. Another notable case takes place when *n*_1_ − *n*_2_ + *n*_3_ = 0. In this case, with both a PLE and an ORE, CORPSE results in

15 $${R}_{{\rm{C}}ORPSE}^{^{\prime} }(\theta ,\phi )={R}^{^{\prime} }({\theta }_{3},\phi ){R}^{^{\prime} }({\theta }_{2},\phi +\pi ){R}^{^{\prime} }({\theta }_{1},\phi )=R(\theta ,\phi )-{\rm{i}}\varepsilon ({\boldsymbol{n}}\cdot {\boldsymbol{\sigma }})R(\theta ,\phi )/2.$$

### Reduced CORPSE in BB1

R-CinBB^15^ is given as follows:

16 $$\begin{array}{rcl}{\theta }_{1} & = & {\theta }_{3}=\pi ,{\theta }_{2}=2\pi ,{\theta }_{4}={\theta }_{6}+2\pi =2\pi +\theta /2-k,{\theta }_{5}=2\pi -2k,\\ {\phi }_{1} & = & {\phi }_{3}=\phi +\arccos [-\theta /(4\pi )],\\ {\phi }_{2} & = & 3{\phi }_{1}-2\phi ,\\ {\phi }_{4} & = & {\phi }_{5}-\pi ={\phi }_{6}=\phi ,\\ k & = & \arcsin [\sin (\theta /2)/2].\end{array}$$

Table 1 shows parameters of *π*/2- and *π*-pulses of the above three composite pulses.

**Table 1:** *π*/2(90°) and *π*(180°) rotation implemented by BB1, CORPSE, and R-CinBB.

Name	Position	*θ* = 90°		*θ* = 180°	
		*θ*/degree	*ϕ*/degree	*θ*/degree	*ϕ*/degree
BB1 (robust for PLEs)	1	180.0	97.2	180.0	104.5
	2	360.0	291.5	360.0	313.4
	3	180.0	97.2	180.0	104.5
	4	90.0	0.0	180.0	0.0
CORPSE (robust for OREs)	1	384.3	0.0	420.0	0.0
	2	318.6	180.0	300.0	180.0
	3	24.3	0.0	60.0	0.0
R-CinBB (robust for both PLEs and OREs)	1	180.0	97.2	180.0	104.5
	2	360.0	291.5	360.0	313.4
	3	180.0	97.2	180.0	104.5
	4	384.3	0.0	420.0	0.0
	5	318.6	180.0	300.0	180.0
	6	24.3	0.0	60.0	0.0

### 300 mM ^13^C-labelled chloroform in acetone-*d*_6_

^13^C-labelled chloroform was purchased from Cambridge Isotopes. To the 300 mM ^13^C-labelled chloroform acetone-*d*_6_ solution, 4 mM of iron(III) acetylacetonate was added. Resulting *T*_1_ (^13^C) and *T*_2_ (^13^C) were ~ 6 s and 200 ms, respectively, while *T*_1_ (^1^H) and *T*_2_ (^1^H) were both ~200 ms.

### 2% HDO in D_2_O

To the solvent-mixture composed of 594 *μ*L of D_2_O and 6 *μ*L of H_2_O, 2 mg of CuCl_2_ was added, resulting in *T*_1_ (^1^H) and *T*_2_ (^1^H) of ~50 ms at 9.7 T. Note that the solvent mixing causes 2 % HDO solution, due to the H-D chemical exchange.

### 300 mM 3-chloro-2,4,5,6-tetrafluoro-benzotrifluoride in benzene-*d*_6_

3-chloro-2,4,5,6-tetrafluoro-benzotrifluoride was diluted with benzene-*d*_6_ to 300 mM solution.

### NMR measurements

All the NMR experiments described in this article were measured on a JNM-ECA500 spectrometer (working at 11.7 T) or JNM-ECZ400S spectrometers (working at 9.4 T) (JEOL RESONANCE Inc.). The 2% HDO sample was measured at 9.4 T (400 MHz for ^1^H), and the other samples at 11.7 T (500 MHz for ^1^H and 488 MHz for ^19^F). The measurements were carried out at 25 °C. A 5 mm ({^1^H, ^19^F}-X) broadband (BB) probe was used (11.7 T), and 5 mm ROYAL probes were used (9.4 T). We took 1/4 of the square 2*π*-pulse duration as the pulse duration of a square *π*/2-pulse (11.7 T). Instead, the nonlinear least square curve fitting method^29^ was used (9.4 T). During the ^13^C observing experiments, ^1^H are decoupled by WALTZ16 decoupling trains. The acquired 2D time-domain data were processed as follows. For both *t*_1_ and *t*_2_ periods, the shifted sine-bell window function was multiplied. For *t*_1_, zero-filling was done once. These data were then Fourier-transformed.
